# Relative Incidence of New-Onset Substance Use Disorders Following Traumatic Brain Injury: A Global Retrospective Multicenter Analysis Using the TriNetX Database

**DOI:** 10.3390/jcm15031182

**Published:** 2026-02-03

**Authors:** Zachary T. Hoglund, Christopher Sollenberger, Kyle W. Scott, John D. Arena, Visish M. Srinivasan, Jan-Karl Burkhardt, Jeffrey Turnbull, Julio Rosado-Philippi, Heather Heitkotter, Alexander I. Helfand, Daniel W. Griepp, Chad F. Claus

**Affiliations:** 1 Department of Neurosurgery, University of Pennsylvania, Philadelphia, PA 19104, USAkscott20@hfhs.org (K.W.S.);; 2 Division of Neurosurgery, Henry Ford Providence Hospital, Michigan State University College of Human Medicine, Southfield, MI 48075, USA; jturnbu2@hfhs.org (J.T.); jrosado1@hfhs.org (J.R.-P.); hheitko1@hfhs.org (H.H.); cclaus2@hfhs.org (C.F.C.); 3 Division of Neurology, Henry Ford Health System, Michigan State University College of Human Medicine, Detroit, MI 48038, USA; ahelfan1@hfhs.org

**Keywords:** traumatic brain injury, trauma, substance abuse disorder, post-traumatic outcome, TriNetX Database

## Abstract

**Background:** Traumatic brain injury (TBI) imposes a substantial public health burden through long-term physical, cognitive, and psychiatric effects. This includes substance use disorders (SUDs) for which TBI is a demonstrated risk factor; however, prior studies have not comprehensively compared relative incidences of SUD subtypes post-TBI or differences between intracranial hemorrhage (ICH) and non-ICH TBI in patients without prior SUD history. This global retrospective analysis using the TriNetX database aims to quantify new-onset SUD incidence post-TBI in the largest cohort of patients evaluated to date, with cohorts stratified by SUD subtype and ICH versus non-ICH TBI, to highlight opportunities for post-injury care models to mitigate SUD risk. **Methods:** De-identified data from the TriNetX Research Network were used to select patients with TBI (*n* = 1,889,112) and define distinct cohorts based upon the presence (*n* = 420,868) or absence (*n* = 1,471,592) of ICH. Patients with previously diagnosed SUD before the date of TBI were excluded. Patient demographics and medical comorbidities were calculated for each group. The incidence of new SUD diagnosis over the lifetime and at 1-, 3-, and 5-years post-TBI were calculated and compared. Subtypes of SUD were defined and calculated based on the specific substance used. Propensity scores were calculated to create balanced matched ICH and non-ICH cohorts (*n* = 331,812 each) were used for comparisons of 5-year SUD incidence. **Results:** In the full TBI cohort, 5-year new SUD incidence was 4.2% overall, with nicotine (2.4%) and alcohol (1.1%) predominating, followed by cannabis (0.9%) and opioids (0.4%). Rates of SUDs increased over time, but attenuated beyond 5 years, with approximately 50% of those who would ultimately be diagnosed with SUD manifesting (lifetime) by 3 years post-TBI. After propensity matching, non-ICH TBI showed higher 5-year risk for any SUD (4.2% vs. 3.6%; risk difference −0.65%, *p* < 0.0001) and all subtypes (*p* < 0.05) except inhalants (*p* = 0.53). **Conclusions:** This largest-to-date analysis of new-onset SUD post-TBI demonstrates significantly higher rates of SUD in TBI patients; rates of nicotine, alcohol, cannabis, and opioid use disorders were most common. Non-ICH TBI patients demonstrated greater rates of SUD after injury than patients with ICH-associated TBI. Of patients suffering from TBI without ICH who would eventually be diagnosed with SUD, approximately 50% had obtained that diagnosis within 3 years of the injury. Taken together, these findings demonstrate the clinical need for routine SUD screening in post-TBI care, especially for 3 years post-injury. Such an intervention has the potential to significantly alleviate the public health burden and associated cost of care for TBI-associated substance use disorder patients.

## 1. Introduction

Traumatic brain injury (TBI) and associated health problems constitute a significant public health burden. In the US, there were over 64,000 TBI-related deaths in 2021 alone, with far more patients requiring care for long-term physical impairment, cognitive dysfunction, and emotional problems associated with TBI [1,2,3,4]. These injuries range from mild concussions to severe traumas associated with intracranial hemorrhage. Etiologies are diverse and include falls, motor vehicle accidents, assaults, sports-related head injuries, and others. In addition to management of organic injury, sequelae can manifest with neurocognitive and psychiatric problems [2]. Unfortunately, both providers and patients do not always recognize and treat these deficits [5]. For patients suffering from post-concussive syndrome, there is an increased rate of substance use disorder (SUD) and this is one condition which may be associated with future morbidity and mortality [6,7,8].

TBI has been demonstrated to precipitate or exacerbate SUDs post-injury, as patients face challenges related to pain, disability, cognition, memory, as well as depression, anxiety, and post-traumatic stress. Neurocognitive measures of executive functioning and impulsivity may also be affected by TBI [2,9,10]. Neurobiological mechanisms underpin the vulnerability to SUD, as TBI has been shown to induce long-lasting dysregulation in dopamine transport at a cellular level [11,12,13,14,15]. Aberrant dopamine trafficking may amount to network dysfunction, as TBI has previously been linked to impaired functionality of mesolimbic and mesocortical circuits [16]. Dysfunction of these networks is a hallmark finding which contributes to addictive behavior in the context of substance use disorder [11,17,18].

While TBI has been established as a risk factor for SUD, there is surprisingly little work specifically characterizing post-TBI risk for SUD as a function of TBI sub-type. Many differences in both mechanism of injury and patient population exist for those who sustain TBI with intracranial hemorrhage (ICH) versus non-ICH TBI; however, prior studies have not extensively compared the relative incidence of new SUD diagnoses post-TBI in a single cohort. Furthermore, prior studies have not explicitly focused on populations without prior SUD history. Addressing these gaps is essential for assessing SUD risk following TBI and determining which TBI patients might benefit from SUD screening and further clinical intervention.

The TriNetX database provides a large-scale, real-world dataset for addressing these gaps through a comprehensive, multicenter repository of patient health records [19,20]. Using the TriNetX database, this study examines new-onset SUD in a population of 1,889,112 TBI patients without previously diagnosed SUD. We examine and compare the incidence of new SUD diagnoses post-TBI, including alcohol, nicotine, cannabis, opioids, cocaine, and others at 1-year, 3-year, 5-year, and lifelong post-TBI periods. By comparing ICH and non-ICH cohorts as well as the relative rate of new SUD diagnoses, we aim to characterize differential SUD risks between TBI subtypes and highlight opportunities for clinical intervention. This work tests the hypothesis that ICH and non-ICH TBI are associated with significantly different rates of SUD incidence after injury.

## 2. Materials and Methods

### 2.1. Study Design and Data Acquisition

This retrospective multicenter cohort study utilized de-identified patient data collected from the TriNetX Research Network (Cambridge, MA, USA), a global health research network providing access to electronic health records and health outcomes data. The platform uses standardized terminologies, including International Classification of Diseases, Tenth Revision (ICD) codes to define and compare patient cohorts. All analyses were conducted within the TriNetX LIVE™ platform, enabling real-time analysis of de-identified, aggregate patient information [21].

### 2.2. Cohort Definition

Three cohorts were defined: (1) TBI not delineated for ICH, (2) TBI patients with ICH, and (3) TBI patients without ICH. The TBI not delineated for ICH cohort included all patients with at least one ICD diagnosis code in category S06 (intracranial injury). The ICH TBI cohort comprised patients with codes for traumatic subdural hemorrhage (S06.5), traumatic subarachnoid hemorrhage (S06.6), or epidural hemorrhage (S06.4). The non-ICH TBI cohort included patients with an S06 diagnosis but excluded those with S06.5, S06.6, or S06.4 codes. For all cohorts, patients were excluded if they had a prior diagnosis of any SUD (ICD F10-F19) on or before the index TBI diagnosis date. The index TBI diagnosis date is the first date a patient meets inclusion criteria for a cohort, and each patient is included only once.

### 2.3. Baseline Characteristics and Unmatched Outcome Analyses

Baseline characteristics (age, sex, race, ethnicity) were calculated for each cohort using TriNetX’s “advanced cohort exploration” tool. Outcome analyses were performed independently for each cohort. Outcomes were defined as new diagnoses of SUDs (ICD F10–F19) stratified by subtype: any SUD, alcohol-related (F10), nicotine-related (F17), cannabis-related (F12), opioid-related (F11), cocaine-related (F14), inhalant-related (F18), hallucinogen-related (F16), sedative/hypnotic/anxiolytic-related (F13), other stimulant-related (F15), and other psychoactive substance-related (F19). Incidence rates were calculated for follow-up periods from 1-day post-TBI to 1 year, 3 years, 5 years, and all-time post-TBI. Rates of any SUD diagnosis were compared between cohorts at each timepoint using two-sample tests of proportions (z-test) in MATLAB R2025b. Baseline and unmatched outcome analyses were completed on 8 November 2025.

### 2.4. Propensity Score-Matched Comparison

To compare SUD incidence between ICH and non-ICH TBI cohorts while controlling for confounders, two 1:1 propensity score-matched analyses were conducted using TriNetX’s “compare outcomes” tool. Matching was based on all baseline characteristics and comorbidities to generate propensity scores; calculation of propensity scores was similar to that previously described by other investigators [22]. Both propensity matched analyses performed were similar, except the second analysis only included patients with Glasgow Coma Scale (GCS) scores on the date of TBI diagnosis and balanced for this characteristic, while the first analysis did not incorporate GCS. Comorbidities were selected a priori based on established risk factors for SUDs, such as mental health disorders (e.g., anxiety (F40–F48), mood disorders (F30–F39)), seizure disorders (G40), sleep disorders (F51, G47), and other conditions which may suggest prior undisclosed substance use (e.g., human immunodeficiency virus (B20), hepatitis C (B17.1, B18.2), and chronic obstructive pulmonary disease (J44)). The primary outcome was the risk of new SUD diagnoses (overall and subgrouped according to substance type) at 5 years post-TBI. Results were reported as risk differences between ICH and non-ICH TBI cohorts with associated *p*-values. Risk difference was defined as ICH minus non-ICH risk. Statistical significance was set at *p* < 0.05 for all comparisons. The non-GCS matched comparison was completed on 8 November 2025, while the GCS-matched analysis was completed on 10 December 2025.

## 3. Results

### 3.1. Patient Demographics

Using the TriNetX database, we identified a cohort of 1,889,112 patients diagnosed with TBI not delineated for ICH who had no prior history of SUDs, and cohorts of 420,868 patients with ICH-associated TBI and 1,471,592 patients with non-ICH TBI. TriNetX returned data from five countries and 108 centers for the TBI not delineated for ICH and ICH-associated TBI cohorts, and 109 centers for the non-ICH TBI cohort. The mean age at the time of TBI diagnosis was 38.6 years (standard deviation (SD): 26.8) for the overall cohort, while patients with ICH-associated TBI were substantially older, with a mean age of 59.9 years (SD: 26.8) compared to 32.5 years (SD: 23.4) for those with non-ICH TBI.

Sex distribution showed a slight male predominance overall (52%) with a higher male proportion in the ICH subgroup (56%) compared to non-ICH (51%). Racial demographics were predominantly white (65%), followed by unknown race (13%), Black or African American (11%), Asian (5%), other race (5%), Native Hawaiian or other Pacific Islander (1%), and American Indian or Alaska Native (<1%). Ethnicity was reported as not Hispanic or Latino in 67% of patients, unknown in 23%, and Hispanic or Latino in 9% (Table 1).

### 3.2. Incidence of New-Onset SUDs in Unmatched Cohorts

**Figure 1:**
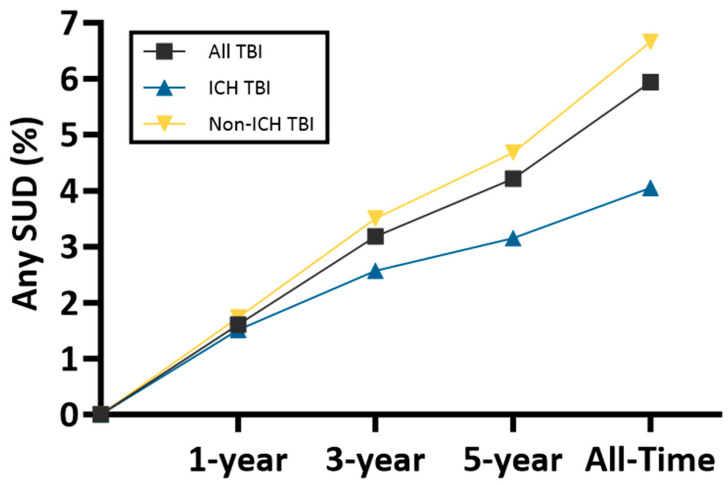
SUD incidence by injury type. Incidence of any SUD across 1-year, 3-year, 5-year, and all-time post-TBI time intervals is plotted. Individual lines are plotted for all TBI (TBI not delineated for ICH), ICH TBI, and non-ICH TBI cohorts.

In an unmatched analysis, the cumulative incidence of new-onset SUDs increased progressively over time across all follow-up periods (1-year, 3-year, 5-year, and all-time post-TBI). At 1-year post-TBI, the incidence of any SUD was 1.607% for any TBI, 1.511% for ICH, and 1.735% for non-ICH TBI. This rose to 3.182%, 2.569%, and 3.500% at 3 years; 4.216%, 3.151%, and 4.680% at 5 years; and 5.942%, 4.054%, and 6.660% over all-time post-TBI for any TBI, ICH TBI, and non-ICH TBI, respectively. Rates of SUD across all measured timepoints significantly differed between the cohorts (*p* < 0.0001) (Figure 1, Table 2).

Comparing timepoints within a cohort, the most pronounced relative increase in the larger cohort of TBI not delineated for ICH patients was observed between years 1 and 3 (98% relative increase; from 1.607% to 3.182%). Thereafter, the accrual of new diagnoses attenuated, with a 32% relative increase from year 3 to year 5, followed by an additional increase approximating 41% thereafter. The non-ICH cohort demonstrated a greater early acceleration (102% relative increase from year 1 to 3) followed by gradual deceleration, in contrast to the ICH cohort, which exhibited a comparatively flatter trajectory across all intervals. Within cohorts, all incidence differences between time intervals were significant (*p* < 0.0001) (Figure 1, Table 2).

Among specific SUD subtypes, nicotine-related disorders were the most common across all time periods for any TBI with a 5-year incidence of 2.369%. Alcohol-related disorders followed, with a 5-year incidence of 1.104%. Cannabis-related disorders had a lower 5-year incidence of 0.868%, while opioid-related disorders had a 5-year incidence of 0.424%. Less common subtypes included cocaine (5-year: 0.168%), inhalant-related disorders (0.082%), hallucinogen-related disorders (0.031%;), sedative/hypnotic/anxiolytic-related disorders (0.122%), other stimulant-related disorders (0.169%), and other psychoactive substance-related disorders (0.494%) (Table 2).

### 3.3. Propensity Score-Matched Analysis

To account for baseline differences between ICH and non-ICH cohorts, propensity score matching was performed on all baseline characteristics and comorbidities associated with SUD risk or which may indicate prior SUD use, yielding balanced cohorts of 331,812 patients each for ICH-associated TBI and non-ICH TBI (Supplementary Table S1). In this matched analysis for 5-year outcomes, the incidence of any SUD was 3.558% (*n* = 11,805) in the ICH group and 4.208% (*n* = 13,963) in the non-ICH group, with a risk difference of −0.65% (*p* < 0.0001).

At 5 years post-TBI, the non-ICH group showed significantly higher risk for nearly all SUD subtypes compared to the ICH group (Table 2). The largest risk differences were for nicotine-related disorders (−0.435%, *p* < 0.0001) and alcohol-related disorders (−0.161%, *p* < 0.0001), followed by cannabis (−0.079%, *p* < 0.0001), other psychoactive substances (−0.066%, *p* < 0.0001), opioid (−0.046%, *p* = 0.0054), cocaine (−0.046%, *p* < 0.0001), sedative/hypnotic/anxiolytic (−0.037%, *p* < 0.0001), other stimulant (−0.023%, *p* = 0.014), and hallucinogen-related disorders (−0.008%, *p* = 0.0314). Inhalant-related disorders showed no significant difference between cohorts (0.004%, *p* = 0.5339) (Table 3).

A second propensity score-matched comparison was conducted including only patients who had GCS scores recorded on the date of TBI diagnosis available in TriNetX (<5% of TBI patients in the TriNetX database). This analysis balanced cohorts based on GCS score in addition to baseline statistics and comorbidities, resulting in cohorts of 13,521 patients each for ICH and non-ICH TBI with GCS scores of 13.1 (SD: 3.54) and 13.9 (SD: 2.89), respectively (Supplementary Table S2). Results were largely similar to the non-GCS matched analysis, with higher rates of SUDs in the non-ICH cohort and a risk difference for any SUD of −1.420% (*p* < 0.0001). Subtype differences were also similarly significant (*p* < 0.05), except for opioid and sedative, hypnotic, or anxiolytic disorders. Inhalant- and hallucinogen-related disorders had incidences too small to be reported by TriNetX (Supplementary Table S3).

## 4. Discussion

This retrospective multicenter study using the TriNetX database investigated new-onset substance use disorders (SUDs) in the largest cohort of TBI patients to date, comprising over 1.8 million individuals. Our findings reveal a progressive increase in SUD incidence post-TBI, with nicotine- and alcohol-related disorders predominating among subtypes. Propensity-matched analyses further demonstrated differential risks by injury type, with non-ICH TBI associated with higher SUD vulnerability compared to traumatic ICH. These observations underscore TBI’s contribution as a risk factor for substance use and highlight opportunities for targeted screening and prevention strategies.

Across the full population of TBI patients, we found a 5-year incidence of new-onset SUDs of any subtype of 4.216%. This rate is somewhat lower than that found in the TBIMs study, which reported a 5-year incidence of illicit drug use of 12% post-TBI [2]. This difference might be attributed to inclusion of patients with prior history of SUD in the TBIMs cohort, which would be expected to inflate incident SUD. Regarding SUD subtype, we found that nicotine-related disorders exhibited the highest incidence at 2.369% (*n* = 44,750), followed by alcohol-related disorders at 1.104% (*n* = 20,865), cannabis-related disorders at 0.868% (*n* = 16,396), and opioid-related disorders at 0.424% (*n* = 8008), with other less common subtypes below 0.2%. This hierarchy underscores a heightened post-TBI vulnerability to more commonly accessible substances like nicotine and alcohol. Prior studies, although limited, have also found higher rates of nicotine and cannabis use relative to opioid use in TBI patients [23]. These findings suggest post-TBI screening for substance use disorder might prioritize identification and treatment of nicotine and alcohol use disorders, as these substances and their associated misuse are more accessible and more prevalent. Integration and routine use of validated screening tools like the Alcohol Use Disorders Identification Test (AUDIT) or Drug Abuse Screening Test (DAST) in follow-up care could facilitate early intervention [24,25].

The timeline with which SUD occurred after TBI is of significant clinical relevance; the greatest increase in SUD incidences was observed between years 1 and 3 post-injury. This nonlinear trajectory suggests the initial 1- to 3-year post-injury interval may constitute the phase of peak vulnerability, during which TBI’s neurocognitive effects coupled with patients regaining adequate functional independence and social access facilitate substance exposure and dependence development. This is supported by analyses which show that patients with pre-existing SUDs exhibit decreased substance use in the first year post-injury but return to pre-injury use levels by year two [26]. Other work by Malec et al. found that patients who had received diagnoses of drug addiction or alcoholism within 10 years after TBI had trajectories of functional independence which were better than those without these diagnoses, suggesting improved function may facilitate greater access to addictive substances [27].

Thus, the accelerated early incidence of SUDs in our data supports the implementation of intensive, recurrent SUD screening particularly in the first 1–3 years post-TBI. The sustained, albeit diminished, elevation in incidence beyond 5 years, contributing an additional 1.7 percentage points in the overall TBI cohort, may further suggest that TBI imparts a chronic, enduring SUD risk rather than a transient one. As such, SUD surveillance may be beneficial for longitudinal care beyond 3 years. However, without a proper control cohort comparison, this is challenging to conclude based solely on these data. Based on these data, screening integration could involve administration of AUDIT/DAST screening protocols at neurosurgical/primary care follow-ups for 3 years post-injury, with multidisciplinary referrals for positive results.

Our propensity-matched analysis between ICH and non-ICH TBI subtypes revealed consistently higher 5-year SUD risks in the non-ICH subgroup across most subtypes, despite ICH often indicating more severe injury with greater disability [28]. In the matched cohorts of 331,812 patients each, the 5-year incidence of any SUD was 4.208% (*n* = 13,963) in the non-ICH group compared to 3.558% (*n* = 11,805) in the ICH group, yielding a significant risk difference of −0.65% (*p* < 0.0001). The non-ICH TBI group also demonstrated a higher risk for all SUD subtypes individually (*p* < 0.05), except for inhalant-related disorders (*p* = 0.5339). After adjusting for GCS score, a significantly higher rate of any SUD in non-ICH TBI was still observed (*p* < 0.0001).

This counterintuitive pattern may reflect that the greater physical, cognitive, and psychosocial impairments associated with traumatic ICH and severe TBI may reduce motivation for use, be associated with increased supervision and support in rehabilitation settings, or limit access to addictive substances. In support of this hypothesis, analyses including patients with prior-substance use have found that substance use decreases modestly after moderate-to-severe TBI, possibly due to increased social isolation and impaired psychosocial adjustment, whereas individuals with milder injury showed no change or heightened rates of substance use post-TBI [26,29,30]. Additionally, as differences remained after matching for GCS at diagnosis, the relative frequency and duration of clinical follow-up for traumatic ICH patients may be more intensive than for non-ICH patients, contributing to different levels of supervision and counseling post-injury.

It is also possible that different injury mechanisms between ICH- and Non-ICH-associated TBI may impart unique long-term sequelae between injury types. For example, non-ICH TBI may more frequently involve diffuse axonal injury (DAI) as a primary injury mechanism, which is a hallmark of acceleration–deceleration trauma characterized by widespread axonal shearing, disruption of axoplasmic transport, and secondary cascades including intracellular calcium dysregulation, neuroinflammatory responses (e.g., via TNF-α, IL-6, and NF-κB pathways), glymphatic system impairment, and oxidative stress [31,32]. It may be possible that these diffuse processes affect mesolimbic and mesocortical circuits, amplifying dopamine dysregulation, impulsivity, and SUD risk relative to more focal contusions and cerebral hypoperfusion-mediated injury seen in ICH-associated TBI [11,31,33]. Despite propensity matching, residual behavioral factors in younger non-ICH patients, such as greater social reintegration and substance access, could further elevate SUD incidence in the context of non-ICH TBI and DAI. However, as the injuries of any TBI are highly varied, it is challenging to conclusively identify injury mechanism as a mediating factor without detailed patient-level data on injury characteristics.

Overall, these disparities emphasize the need for tailored SUD prevention in non-ICH TBI patients, where more mild disability, less extensive post-TBI monitoring, and perhaps more diffuse injury mechanisms may allow for increased SUD risk.

### Limitations

Our study has several limitations inherent to its retrospective design and data source. The cohorts rely on ICD-10 codes for TBI and SUD definitions, which may not fully capture mild cases or undocumented substance use, leading to conservative incidence estimates. ICH/non-ICH stratification serves as a proxy for severity but lacks granularity on injury mechanisms, injury severity, or neuroimaging details, potentially confounding subtype comparisons and conclusions. TriNetX, while offering a large, multicenter repository of de-identified electronic health records, introduces selection bias toward patients with documented healthcare encounters, possibly overrepresenting severe cases or those in integrated systems and underrepresenting underserved populations [19]. Additionally, the TriNetX population may not accurately reflect SUD incidence in the general population, limiting generalizability. While propensity matching adjusting for baseline characteristics was performed to compare ICH and non-ICH cohorts, other variables not accounted for may influence cohort comparability. Uncaptured variables include injury severity details beyond GCS, polytrauma, treatments, lesion location, neurological deficits, and psychosocial factors (e.g., social support), which may mediate SUD risk via chronic pain or social isolation and may be areas for future research. Additionally, the supplementary GCS-matched sub-analysis, while confirming higher SUD risks in non-ICH TBI, is restricted to a small subset (*n* = 13,521 per cohort) and may not fully represent the broader population. Missing GCS data could thus attenuate observed differences if undocumented severe cases in non-ICH TBI inflate SUD incidence, or conversely, if ICH cases with missing low GCS scores are underrepresented. Confounding among matched characteristics is not accounted for in these analyses as well, limiting generalization for patients with multiple versus single pre-injury comorbidities. Part and partial correlations could evaluate these limitations but are not available in TriNetX propensity-matching. Future prospective studies with more detailed inclusion criteria could address these gaps, enhancing generalizability and mechanistic insights.

Despite these limitations, our findings demonstrate a clinically significant burden of substance use disorders among TBI patients in the years following injury. These data support improved SUD screening in TBI follow-up care, focusing on alcohol, nicotine, cannabis, and opioid use disorders. Additionally, our results finding increased SUD risk in non-ICH versus ICH-associated TBI serve to generate hypotheses for future investigation into the effect of TBI severity, injury mechanisms, and treatment modalities on post-TBI SUD risk.

## 5. Conclusions

This largest-to-date analysis of over 1.8 million TBI patients without prior SUD history demonstrates a significant, progressive incidence of new-onset SUDs 5-years post-TBI, with nicotine and alcohol disorders predominating followed by cannabis and opioids. Additionally, this analysis found that non-ICH-associated TBI confers significantly higher risks across most SUD subtypes relative to traumatic ICH. These findings highlight TBI’s substantial contribution to addiction pathology and supports the widespread adoption of routine, subtype-specific SUD screening in post-TBI care to enable early clinical intervention, particularly up to 3 years post-TBI. By evaluating differential risks between TBI subtypes, our results support tailored SUD screening particularly for non-ICH TBI patients, who may face greater opportunities for substance initiation post-TBI due to less disabling impairments and less monitoring compared to traumatic ICH patients. These data support the implementation of strategies to mitigate the public health burdens associated with traumatic brain injury and substance use disorder.

## Figures and Tables

**Table 1:** Baseline characteristics.

Variable	Any TBI	ICH	Non-ICH
Total Patients, *n*	1,889,112	420,868	1,471,592
Age at TBI, mean (SD)	38.6 (26.8)	59.9 (26.8)	32.5 (23.4)
Sex, *n* (%)	-	-	-
Male	983,220 (52%)	237,490 (56%)	748,180 (51%)
Female	904,818 (48%)	183,129 (44%)	722,586 (49%)
Unknown	1074 (<1%)	249 (<1%)	826 (<1%)
Race, *n* (%)	-	-	-
White	1,218,738 (65%)	277,738 (66%)	943,264 (64%)
Unknown Race	241,537 (13%)	48,690 (12%)	193,041 (13%)
Black or African American	209,158 (11%)	36,928 (9%)	172,749 (12%)
Asian	103,123 (5%)	34,825 (8%)	68,484 (5%)
Other Race	94,899 (5%)	17,753 (4%)	77,273 (5%)
Native Hawaiian or Other Pacific Islander	12,263 (1%)	2457 (1%)	9837 (1%)
American Indian or Alaska Native	9394 (<1%)	2477 (1%)	6944 (<1%)
Ethnicity, *n* (%)	-	-	-
Not Hispanic or Latino	1,269,214 (67%)	288,554 (69%)	983,245 (67%)
Unknown Ethnicity	443,112 (23%)	99,014 (24%)	344,564 (23%)
Hispanic or Latino	176,786 (9%)	33,300 (8%)	143,783 (10%)

**Table 2:** Relative incidence of substance use disorders.

Incidence, *n* (%)	1-Year			3-Year			5-Year			All Time		
	Any TBI *n* = 1,889,112	ICH *n* = 420,868	Non-ICH *n* = 1,471,592	Any TBI *n* = 1,889,112	ICH *n* = 420,868	Non-ICH *n* = 1,471,592	Any TBI *n* = 1,889,112	ICH *n* = 420,868	Non-ICH *n* = 1,471,592	Any TBI *n* = 1,889,112	ICH *n* = 420,868	Non-ICH *n* = 1,471,592
Any SUD (F10-F19)	30,366 (1.607%)	6361 (1.511%)	25,531 (1.735%)	60,119 (3.182%)	10,813 (2.569%)	51,509 (3.500%)	79,642 (4.216%)	13,262 (3.151%)	68,865 (4.68%)	112,243 (5.942%)	17,060 (4.054%)	98,001 (6.66%)
Alcohol (F10)	7890 (0.418%)	1969 (0.468%)	6393 (0.434%)	15,629 (0.827%)	3369 (0.800%)	12,953 (0.880%)	20,865 (1.104%)	4191 (0.996%)	17,476 (1.188%)	31,369 (1.661%)	5671 (1.347%)	26,699 (1.814%)
Nicotine (F17)	16,234 (0.859%)	3229 (0.767%)	13,752 (0.934%)	33,035 (1.749%)	5650 (1.342%)	28,515 (1.938%)	44,750 (2.369%)	7048 (1.675%)	39,019 (2.651%)	65,698 (3.478%)	9378 (2.228%)	57,917 (3.936%)
Cannabis (F12)	4840 (0.256%)	672 (0.160%)	4390 (0.298%)	11,369 (0.602%)	1245 (0.296%)	10,501 (0.714%)	16,396 (0.868%)	1622 (0.385%)	15,218 (1.034%)	25,492 (1.349%)	2397 (0.570%)	23,702 (1.611%)
Opioid (F11)	2608 (0.138%)	632 (0.150%)	2124 (0.144%)	5748 (0.304%)	1200 (0.285%)	4782 (0.325%)	8008 (0.424%)	1555 (0.369%)	6734 (0.458%)	12,948 (0.685%)	2181 (0.518%)	11,144 (0.757%)
Cocaine (F14)	1034 (0.055%)	175 (0.042%)	914 (0.062%)	2223 (0.118%)	321 (0.076%)	1993 (0.135%)	3168 (0.168%)	425 (0.101%)	2851 (0.194%)	5507 (0.292%)	679 (0.161%)	4998 (0.340%)
Inhalant-related disorders (F18)	551 (0.029%)	100 (0.024%)	484 (0.033%)	1173 (0.062%)	182 (0.043%)	1046 (0.071%)	1547 (0.082%)	228 (0.054%)	1389 (0.094%)	2087 (0.110%)	298 (0.071%)	1883 (0.131%)
Hallucinogen-related disorders (F16)	174 (0.009%)	20 (0.005%)	162 (0.011%)	397 (0.021%)	46 (0.011%)	366 (0.025%)	592 (0.031%)	62 (0.015%)	548 (0.037%)	970 (0.051%)	100 (0.024%)	900 (0.061%)
Sedative, hypnotic, or anxiolytic -related disorders (F13)	803 (0.043%)	175 (0.042%)	678 (0.046%)	1667 (0.088%)	304 (0.072%)	1441 (0.098%)	2313 (0.122%)	410 (0.097%)	1992 (0.135%)	3628 (0.192%)	589 (0.140%)	3162 (0.215%)
Other stimulant- related disorders (F15)	1022 (0.054%)	198 (0.047%)	887 (0.060%)	2206 (0.117%)	352 (0.084%)	1949 (0.132%)	3189 (0.169%)	462 (0.110%)	2849 (0.194%)	5745 (0.304%)	729 (0.173%)	5205 (0.354%)
Other psychoactive substance-related disorders (F19)	3055 (0.262%)	554 (0.132%)	2661 (0.181%)	6663 (0.353%)	1020 (0.242%)	5888 (0.400%)	9327 (0.494%)	1325 (0.315%)	8308 (0.565%)	14,819 (0.784%)	1934 (0.460%)	13,335 (0.906%)

**Table 3:** Propensity score-matched analysis.

Incidence, *n* (% Risk)	ICH TBI *n* = 331,812	Non-ICH TBI *n* = 331,812	Risk Difference	*p*-Value
Any SUD	11,805 (3.558%)	13,963 (4.208%)	−0.650%	<0.0001
Alcohol	3711 (1.118%)	4246 (1.280%)	−0.161%	<0.0001
Nicotine	6250 (1.884%)	7694 (2.319%)	−0.435%	<0.0001
Cannabis	1552 (0.468%)	1814 (0.547%)	−0.079%	<0.0001
Opioid	1442 (0.435%)	1595 (0.481%)	−0.046%	0.0054
Cocaine	412 (0.124%)	565 (0.170%)	−0.046%	<0.0001
Inhalant-related disorders	225 (0.068%)	212 (0.064%)	0.004%	0.5339
Hallucinogen-related disorders	60 (0.018%)	86 (0.026%)	−0.008%	0.0314
Sedative, hypnotic, or anxiolytic-related disorders	364 (0.11%)	486 (0.146%)	−0.037%	<0.0001
Other stimulant-related disorders	441 (0.133%)	517 (0.156%)	−0.023%	0.014
Other psychoactive substance-related disorders	1222 (0.368%)	1441 (0.434%)	−0.066%	<0.0001

## Data Availability

Restrictions apply to the availability of these data. Data were obtained from the TriNetX Research Network and are available from TriNetX for researchers who meet TriNetX’s criteria for access. The data underlying this article cannot be shared publicly, but can be accessed by valid TriNetX license holders or upon reasonable request and with the permission of TriNetX.

## Supplementary Materials

The following supporting information can be downloaded at: https://www.mdpi.com/article/10.3390/jcm15031182/s1, Table S1: Baseline Characteristics After Propensity Matching for Non-GCS Matched Analysis; Table S2: Matched Characteristics and Comorbidities for GCS-Matched Analysis; Table S3: ICH vs. non-ICH TBI comparison by substance abuse disorder subgroups.

## Abbreviations

TBI	Traumatic Brain Injury
SUD	Substance Use Disorder
ICH	Intracranial Hemorrhage
AUDIT	Alcohol Use Disorders Identification Test
DAST	Drug Abuse Screening Test
