# Supplementary material for: Relative Incidence of New-Onset Substance Use Disorders Following Traumatic Brain Injury: A Global Retrospective Multicenter Analysis Using the TriNetX Database
Source: J Clin Med. 2026 Feb 3;15(3):1182. doi: 10.3390/jcm15031182 (PMC12897771; doi:10.3390/jcm15031182)
Supplement: Supplementary file 1 [file jcm-15-01182-s001.zip › Supplementary Table S1.pdf]

Table S1. Baseline Characteristics After Propensity Matching for Non-GCS Matched Analysis.

ICH TBI (N = 331,812) and Non-ICH TBI (N = 331,812) characteristics after propensity score matching

Demographics

Cohort			Mean ± SD	Patients	% of Cohort	P-Value	Std diff.
1<br>2	AI	Age at Index	55.3 +/- 27.3<br>54.9 +/- 26.4	331,812<br>331,812	100%<br>100%	<0.001	0.013
1<br>2	F	Female		150,842<br>148,222	45.5%<br>44.7%	<0.001	0.016
1<br>2	2054-5	Black or African American		32,076<br>31,875	9.7%<br>9.6%	0.403	0.002
1<br>2	M	Male		180,785<br>183,389	54.5%<br>55.3%	<0.001	0.016
1<br>2	2106-3	White		221,612<br>216,137	66.8%<br>65.1%	<0.001	0.035
1<br>2	1002-5	American Indian or Alaska Native		1,985<br>2,196	0.6%<br>0.7%	0.001	0.008
1<br>2	UNK	Unknown Race		38,210<br>43,076	11.5%<br>13.0%	<0.001	0.045
1<br>2	2076-8	Native Hawaiian or Other Pacific Islander		2,069<br>2,081	0.6%<br>0.6%	0.852	<0.001
1<br>2	UN	Unknown Ethnicity		79,862<br>84,801	24.1%<br>25.6%	<0.001	0.034
1<br>2	2186-5	Not Hispanic or Latino		223,223<br>217,455	67.3%<br>65.5%	<0.001	0.037
1<br>2	2135-2	Hispanic or Latino		28,727<br>29,556	8.7%<br>8.9%	<0.001	0.009
1<br>2	2131-1	Other Race		15,375<br>15,570	4.6%<br>4.7%	0.256	0.003
1<br>2	2028-9	Asian		20,485<br>20,877	6.2%<br>6.3%	0.047	0.005

Diagnosis

Cohort			Mean ± SD	Patients	% of Cohort	P-Value	Std diff.
1<br>2	B20-B20	Human immunodeficiency virus [HIV] disease (B20)		638<br>585	0.2%<br>0.2%	0.129	0.004
1<br>2	B17.1	Acute hepatitis C		285<br>289	0.1%<br>0.1%	0.867	<0.001
1<br>2	B18.2	Chronic viral hepatitis C		1,212<br>1,170	0.4%<br>0.4%	0.389	0.002
1<br>2	K70	Alcoholic liver disease		591<br>658	0.2%<br>0.2%	0.058	0.005
1<br>2	J44	Other chronic obstructive pulmonary disease		15,091<br>16,231	4.5%<br>4.9%	<0.001	0.016
1<br>2	J43	Emphysema		4,648<br>4,993	1.4%<br>1.5%	<0.001	0.009
1<br>2	F20-F29	Schizophrenia, schizotypal, delusional, and other non-mood psychotic disorders		5,629<br>5,785	1.7%<br>1.7%	0.141	0.004
1<br>2	F33	Major depressive disorder, recurrent		7,703<br>9,241	2.3%<br>2.8%	<0.001	0.029

1<br>2	F31	Bipolar disorder	4,039<br>4,116	1.2%<br>1.2%	0.391	0.002
1<br>2	F43.1	Post-traumatic stress disorder (PTSD)	2,704<br>4,060	0.8%<br>1.2%	<0.001	0.041
1<br>2	G47	Sleep disorders	42,349<br>42,913	12.8%<br>12.9%	0.039	0.005
1<br>2	R52	Pain, unspecified	14,616<br>13,981	4.4%<br>4.2%	<0.001	0.009
1<br>2	F32	Depressive episode	40,624<br>38,123	12.2%<br>11.5%	<0.001	0.023
1<br>2	F41	Other anxiety disorders	41,187<br>38,771	12.4%<br>11.7%	<0.001	0.022
1<br>2	G40	Epilepsy and recurrent seizures	16,564<br>18,522	5.0%<br>5.6%	<0.001	0.026
1<br>2	F51	Sleep disorders not due to a substance or known physiological condition	5,753<br>5,630	1.7%<br>1.7%	0.245	0.003
1<br>2	G89	Pain, not elsewhere classified	47,471<br>48,915	14.3%<br>14.7%	<0.001	0.012
1<br>2	M54	Dorsalgia	71,022<br>69,242	21.4%<br>20.9%	<0.001	0.013
1<br>2	F40-F48	Anxiety, dissociative, stress-related, somatoform and other nonpsychotic mental disorders	49,878<br>47,947	15.0%<br>14.5%	<0.001	0.016
1<br>2	F30-F39	Mood [affective] disorders	47,516<br>46,421	14.3%<br>14.0%	<0.001	0.009
