# Supplementary material for: Relative Incidence of New-Onset Substance Use Disorders Following Traumatic Brain Injury: A Global Retrospective Multicenter Analysis Using the TriNetX Database
Source: J Clin Med. 2026 Feb 3;15(3):1182. doi: 10.3390/jcm15031182 (PMC12897771; doi:10.3390/jcm15031182)
Supplement: Supplementary file 1 [file jcm-15-01182-s001.zip › Supplementary Table S2.pdf]

Supplementary Table S2. Matched Characteristics and Comorbidities for GCS-Matched Analysis.

Propensity score density function - Before and after matching (cohort 1 - purple, cohort 2 - green)

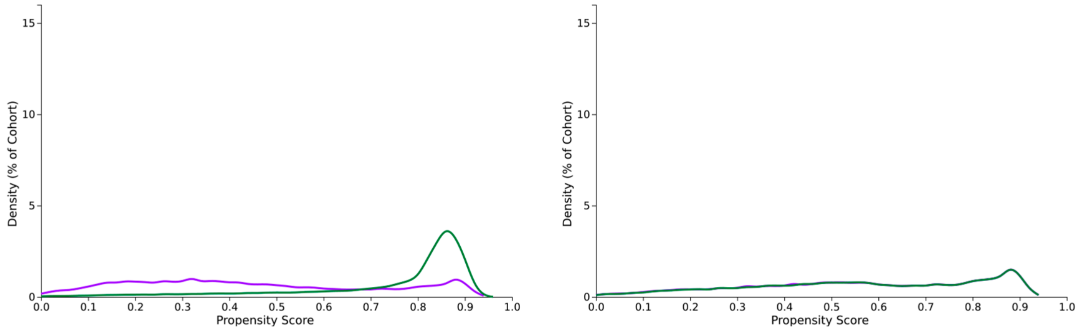

Cohort 1: ICH TBI (N = 13,521) and cohort 2: Non-ICH TBI (N = 13,521) characteristics after propensity score matching

Demographics

Cohort			Mean ± SD	Patients	% of Cohort	P-Value	Std diff.
1<br>2	AI	Age at Index	41.4 +/- 31.5<br>42.5 +/- 28.7	13,521<br>13,521	100%<br>100%	0.004	0.035
1<br>2	F	Female		5,564<br>5,818	41.2%<br>43.0%	0.002	0.038
1<br>2	2054-5	Black or African American		1,021<br>1,005	7.6%<br>7.4%	0.712	0.004
1<br>2	M	Male		7,890<br>7,636	58.4%<br>56.5%	0.002	0.038
1<br>2	2106-3	White		8,771<br>8,751	64.9%<br>64.7%	0.799	0.003
1<br>2	1002-5	American Indian or Alaska Native		556<br>556	4.1%<br>4.1%	1	<0.001
1<br>2	UNK	Unknown Race		1,716<br>1,736	12.7%<br>12.8%	0.716	0.004
1<br>2	2076-8	Native Hawaiian or Other Pacific Islander		37<br>58	0.3%<br>0.4%	0.031	0.026
1<br>2	UN	Unknown Ethnicity		1,377<br>1,476	10.2%<br>10.9%	0.050	0.024
1<br>2	2186-5	Not Hispanic or Latino		9,477<br>9,407	70.1%<br>69.6%	0.354	0.011
1<br>2	2135-2	Hispanic or Latino		2,667<br>2,638	19.7%<br>19.5%	0.657	0.005
1<br>2	2131-1	Other Race		1,027<br>976	7.6%<br>7.2%	0.236	0.014
1<br>2	2028-9	Asian		393<br>439	2.9%<br>3.2%	0.105	0.020

Diagnosis

Cohort			Mean ± SD	Patients	% of Cohort	P-Value	Std diff.
1<br>2	B20-B20	Human immunodeficiency virus [HIV] disease (B20)		13<br>19	0.1%<br>0.1%	0.289	0.013
1<br>2	B17.1	Acute hepatitis C		10<br>10	0.1%<br>0.1%	1	<0.001

Cohort	Code	Description	Mean ± SD	Patients	% of Cohort	P-Value	Std diff.
1<br>2	B18.2	Chronic viral hepatitis C		35<br>35	0.3%<br>0.3%	1	<0.001
1<br>2	K70	Alcoholic liver disease		19<br>15	0.1%<br>0.1%	0.492	0.008
1<br>2	J44	Other chronic obstructive pulmonary disease		464<br>465	3.4%<br>3.4%	0.973	<0.001
1<br>2	J43	Emphysema		190<br>176	1.4%<br>1.3%	0.461	0.009
1<br>2	F20-F29	Schizophrenia, schizotypal, delusional, and other non-mood psychotic disorders		157<br>175	1.2%<br>1.3%	0.320	0.012
1<br>2	F33	Major depressive disorder, recurrent		176<br>200	1.3%<br>1.5%	0.213	0.015
1<br>2	F31	Bipolar disorder		161<br>183	1.2%<br>1.4%	0.233	0.015
1<br>2	F43.1	Post-traumatic stress disorder (PTSD)		141<br>182	1.0%<br>1.3%	0.022	0.028
1<br>2	G47	Sleep disorders		1,165<br>1,288	8.6%<br>9.5%	0.009	0.032
1<br>2	R52	Pain, unspecified		430<br>506	3.2%<br>3.7%	0.011	0.031
1<br>2	F32	Depressive episode		1,277<br>1,427	9.4%<br>10.6%	0.002	0.037
1<br>2	F41	Other anxiety disorders		1,223<br>1,346	9.0%<br>10.0%	0.011	0.031
1<br>2	G40	Epilepsy and recurrent seizures		909<br>969	6.7%<br>7.2%	0.151	0.017
1<br>2	F51	Sleep disorders not due to a substance or known physiological condition		99<br>123	0.7%<br>0.9%	0.106	0.020
1<br>2	G89	Pain, not elsewhere classified		1,885<br>2,136	13.9%<br>15.8%	<0.001	0.052
1<br>2	M54	Dorsalgia		2,684<br>3,023	19.9%<br>22.4%	<0.001	0.061
1<br>2	F40-F48	Anxiety, dissociative, stress-related, somatoform and other nonpsychotic mental disorders		1,592<br>1,767	11.8%<br>13.1%	0.001	0.039
1<br>2	F30-F39	Mood [affective] disorders		1,484<br>1,662	11.0%<br>12.3%	0.001	0.041
**Laboratory**							
Cohort			Mean ± SD	Patients	% of Cohort	P-Value	Std diff.
1<br>2	9269-2	Glasgow coma score total	13.1 +/- 3.5<br>13.9 +/- 2.9	13,521<br>13,521	100%<br>100%	<0.001	0.225
