# Supplementary material for: Relative Incidence of New-Onset Substance Use Disorders Following Traumatic Brain Injury: A Global Retrospective Multicenter Analysis Using the TriNetX Database
Source: J Clin Med. 2026 Feb 3;15(3):1182. doi: 10.3390/jcm15031182 (PMC12897771; doi:10.3390/jcm15031182)
Supplement: Supplementary file 1 [file jcm-15-01182-s001.zip › Supplementary Table S3.pdf]

Table S3. ICH vs. non-ICH TBI comparison by substance abuse disorder subgroups.

Incidence, n (% risk)	**ICH TBI**<br>n = 13,521	**Non-ICH TBI**<br>n = 13,521	**Risk Difference**	**P-value**
**Any SUD**	**446 (3.299%)**	**638 (4.719%)**	**-1.420%**	**<0.0001**
Alcohol	130 (1.107%)	193 (1.427%)	-0.466%	0.0004
Nicotine	242 (1.790%)	358 (2.648%)	-0.858%	<0.0001
Cannabis	76 (0.562%)	120 (0.888%)	-0.325%	0.0016
Opioid	55 (0.407%)	71 (0.533%)	-0.126%	0.1305
Cocaine	14 (0.104%)	27 (0.200%)	-0.095%	0.0422
Inhalant related disorders	*	*	*	*
Hallucinogen related disorders	*	*	*	*
Sedative, hypnotic, or anxiolytic related disorders	13 (0.096%)	15 (0.111%)	-0.015%	0.7053
Other stimulant related disorders	21 (0.155%)	46 (0.340%)	-0.185%	0.0022
Other psychoactive substance related disorders	33 (0.244%)	59 (0.436%)	-0.192%	0.0066

*Incidence of SUD type was too small to be displayed by TriNetX.
